# Integration of the Cortical Haemodynamic Response Measured by Functional Near-Infrared Spectroscopy and Amino Acid Analysis to Aid in the Diagnosis of Major Depressive Disorder

**DOI:** 10.3390/diagnostics11111978

**Published:** 2021-10-25

**Authors:** Samantha K. Ong, Syeda F. Husain, Hai Ning Wee, Jianhong Ching, Jean-Paul Kovalik, Man Si Cheng, Herbert Schwarz, Tong Boon Tang, Cyrus S. Ho

**Affiliations:** 1 Department of Psychological Medicine, National University Health System, Singapore 119228, Singapore; samantha.ong@u.nus.edu; 2 Institute for Health Innovation and Technology (iHealthtech), National University of Singapore, Singapore 119276, Singapore; fabeha@nus.edu.sg; 3 Cardiovascular and Metabolic Disorders Programme, Duke-NUS Graduate Medical School, Singapore 169609, Singapore; haining.wee@duke-nus.edu.sg (H.N.W.); jianhong.ching@duke-nus.edu.sg (J.C.); jean-paul.kovalik@duke-nus.edu.sg (J.-P.K.); 4 Department of Physiology, Yong Loo Lin School of Medicine, National University of Singapore, Singapore 117593, Singapore; phscms@nus.edu.sg (M.S.C.); phssh@nus.edu.sg (H.S.); 5 Centre for Intelligent Signal and Imaging Research (CISIR), University Teknologi PETRONAS, Bandar Seri Iskandar 32610, Perak, Malaysia; tongboon.tang@utp.edu.my; 6 Department of Psychological Medicine, Yong Loo Lin School of Medicine, National University of Singapore, Singapore 119228, Singapore

**Keywords:** diagnosis, near-infrared spectroscopy, verbal fluency test, haemodynamic response, liquid chromatography mass spectrometry, major depressive disorder

## Abstract

Background: Major depressive disorder (MDD) is a debilitating condition with a high disease burden and medical comorbidities. There are currently few to no validated biomarkers to guide the diagnosis and treatment of MDD. In the present study, we evaluated the differences between MDD patients and healthy controls (HCs) in terms of cortical haemodynamic responses during a verbal fluency test (VFT) using functional near-infrared spectroscopy (fNIRS) and serum amino acid profiles, and ascertained if these parameters were correlated with clinical characteristics. Methods: Twenty-five (25) patients with MDD and 25 age-, gender-, and ethnicity-matched HCs were recruited for the study. Real-time monitoring of the haemodynamic response during completion of a VFT was quantified using a 52-channel NIRS system. Serum samples were analysed and quantified by liquid chromatography-mass spectrometry for amino acid profiling. Receiver-operating characteristic (ROC) curves were used to classify potential candidate biomarkers. Results: The MDD patients had lower prefrontal and temporal activation during completion of the VFT than HCs. The MDD patients had lower mean concentrations of oxy-Hb in the left orbitofrontal cortex (OFC), and lower serum histidine levels. When the oxy-haemoglobin response was combined with the histidine concentration, the sensitivity and specificity of results improved significantly from 66.7% to 73.3% and from 65.0% to 90.0% respectively, as compared to results based only on the NIRS response. Conclusions: These findings demonstrate the use of combination biomarkers to aid in the diagnosis of MDD. This technique could be a useful approach to detect MDD with greater precision, but additional studies are required to validate the methodology.

## 1. Introduction

Major depressive disorder (MDD) is a debilitating condition with increasing prevalence and a devastating socioeconomic impact worldwide [1,2]. It is characterised by depressed mood, anhedonia, disturbed sleep and appetite, and anxiety. These attributes are often accompanied with impairments to cognitive and executive functions and working memory, as well as a reduced attention span [3,4]. At least 300 million people globally are currently suffering from depression [5]. At present, depression is the second leading contributor to the global disease burden, but it is predicted to rise to a first-place ranking by 2030 [6]. Without intervention, it can have severe consequences, including an increased risk of developing cardiovascular diseases and suicidal tendencies [7,8]. Current diagnostic procedure and treatment of MDD is based purely on patient history and self-reported symptoms, which are subjective and dependent on individual clinical judgement. The inadequate inter-rater reliability of clinical interviews is further complicated by the heterogeneity of the disorder, and the lack of suitable animal models makes the search for a clear single biomarker onerous [9,10]. There are also no clinically available laboratory or imaging tests that can aid in diagnosis. However, mounting evidence of functional neuroimaging in psychiatry research and multiple dysregulated contributing factors have provided renewed hope for the discovery of novel biosignatures. A greater understanding of the background pathophysiology of the disorder, alongside with a reliable and defined diagnostic approach will provide clinicians with defined guidelines to better tailor treatment plans according to the best possible evidence regarding the effectiveness and tolerability of each drug for each patient. This warrants the development of diagnostic and therapeutic technologies in the field of psychiatry.

Functional near-infrared spectroscopy (fNIRS) is an emerging neuroimaging technique that is non-invasive and complements functional magnetic resonance imaging (fMRI) [11]. Due to its high transmissivity through biological tissues, changes in oxygenated haemoglobin (oxy-Hb) and deoxygenated haemoglobin (deoxy-Hb) in the cerebral cortex can be monitored in real time [12]. The relationship between regional neuron activity and changes in cerebral blood flow and oxygen consumption can be described by a phenomenon known as “neurovascular coupling”. During brain activation, the integration of increased perfusion and higher oxygen consumption results in a net increase in blood volume and oxy-Hb, which can be observed and interpreted using NIRS signals [13,14]. Cerebral activation is determined by the variation in oxy-Hb due to greater increases in blood volume and oxy-Hb as compared with deoxy-Hb [15]. As compared with conventional neuroimaging methods, NIRS is a portable and inexpensive device for neuroimaging that uses infrared light to provide a quantitative measure of haemoglobin changes. It does not involve ionising radiation or severe motion restrictions to give accurate measurements and overcomes the occurrence of claustrophobia, unlike conventional methods such as positron emission tomography (PET) and fMRI [16,17]. Studies have found brain abnormalities in psychiatric patients [18,19,20], with lower activation of the prefrontal cortex (PFC) and temporal lobes [21,22] during cognitive and executive tasks [23]. The PFC is the primary site of behaviour regulation in the brain and is primarily responsible for executive control and emotion regulation, whereas the temporal lobe is highly associated with memory skills. Thus, a reduced haemodynamic response in the frontotemporal cortices may correspond with cognitive impairment and bring about neurophysiological changes associated with depression.

In recent years, metabolomics, particularly amino acids, have been recognised as a promising approach for disease diagnosis and prevention following genomics, transcriptomics, and proteomics, and it now plays a vital role in determining the pathophysiology of depressive disorders [24,25,26,27,28]. The monoamine hypothesis of mood disorders predicts that the development of depression is due to the depletion of corresponding neurotransmitters and neuromodulators in the central nervous system (CNS). Several studies have reported abnormalities in neurotransmitter systems [29,30,31,32,33] and illustrated the relationships among multiple systems in depression [34,35,36]. In fact, antidepressants, such as iproniazid and reserpine, target monoamine depletion at the neuronal synapse and enhance the monoaminergic systems [37]. Amongst all metabolomes, amino acids have been identified as an ideal potential biosignature candidate for disease prediction, given their crucial roles in the homeostasis response and downstream signaling pathways [38]. Norepinephrine and serotonin are well-known monoamines involved in major depression that are synthesised from their amino acid precursors. The use of amino acid therapies to alleviate mood disorders has been popularised in recent years due to its efficacy and precision [39,40,41], and amino acid supplements have also been found to reduce depressive symptoms, as they are converted to neurotransmitters which, in turn, alleviate depressive symptoms [42]. Furthermore, amino acids are a possible candidate for the modulation of neurotransmission and cognitive performance, as they are capable of distinguishing between psychiatric disorders [43,44] and healthy individuals [45,46] with high reliability, making them the best candidate for disease prediction. Although acylcarnitines and lipids have also been implicated in cranial metabolic functions and the development of depression [47,48], few compounds have been reported to differ in patients with MDD as compared with healthy controls (HCs). Cerebral levels are dependent on the concentration of amino acids in the circulation and their ability to cross the blood–brain barrier (BBB) via specialised carrier-mediated mechanisms to support proper CNS functioning [49]. Changes in the corresponding plasma concentration can lead to increased susceptibility to the development of psychiatric disorders and may influence treatment outcomes [50], making amino acids the optimal choice of study for major depressive disorder.

Despite evidence suggesting an interrelation between MDD and biological system abnormalities, there is no single quantitative and non-invasive assessment to aid in the diagnosis and predict the disease trajectory of MDD. Strong biomarker candidates such as brain-derived neurotrophic factor (BDNF) and transcription factor 4 (TCF4) gene variants are unsuitable due to their involvement in other major psychiatric disorders. TCF4 is involved in the schizophrenia network, and BDNF may better reflect a greater level of vulnerability to neurobiological disorders than MDD [51]. Furthermore, BDNF is expressed throughout the nervous system and is less likely to pinpoint impairments only in the CNS [52]. The goal of this study is to, therefore, investigate the use of functional NIRS and amino acids as biomarkers specific to MDD as well as the feasibility of combining both markers to aid in diagnosis. Haemodynamic responses evoked during the VFT and concentrations of 17 amino acids from our laboratory’s amino acid panel were evaluated and compared between MDD patients and HCs. We hypothesised that MDD patients would have less cortical activation in their frontotemporal cortices and decreased amino acid levels as compared with HCs. The sensitivity and specificity of classifying patients with MDD and HCs were based on the magnitude of cortical activity and amino acid profiles, which determined the viability of combination biomarkers for use in MDD diagnosis.

## 2. Materials and Methods

### 2.1. Participants

Twenty-five MDD patients and 25 HCs aged between 21 to 50 were included in the study. Subjects were matched for age, gender, and ethnicity. All participants were right-handed and were native English speakers. Patients were recruited from the outpatient clinic population at the National University Hospital, Singapore, and diagnosis of MDD was made through structured clinical interviews, according to the criteria in the fifth edition of the *Diagnostic and Statistical Manual of Mental Disorders* (DSM-5), by a psychiatrist. Patients screened eligible were immediately recruited for the study. Therefore, we were unable to standardise the time of sample collection and subjects’ diets. Healthy subjects were recruited from the general population and were excluded if they reported a history of psychiatric disorders and/or had received psychotherapy services. Individuals were excluded from the study if they had neurological and/or medical disorders that could affect the CNS, including traumatic brain injuries, cerebrovascular diseases, respiratory diseases, cardiovascular diseases, kidney diseases, hepatic diseases, cancer, epilepsy, substance-related disorders, or mental retardation. Patients with comorbidities inclusive of other major psychiatric disorders (e.g., schizophrenia and bipolar disorders) were also excluded from the study. Depressive symptoms and severity of disease were evaluated for each patient using the 17-item Hamilton Rating Scale for Depression (HAM-D). Healthy subjects with HAM-D scores of 8 and above were also excluded [53]. The study details were fully explained to eligible subjects before they were invited to give written informed consent. The study was performed using the ethical principles presented in the Belmont Report and according to the Declaration of Helsinki. The study was approved by the Domain Specific Review Board of the National Healthcare Group, Singapore (protocol number 2019/00141).

### 2.2. Verbal Fluency Test

**Figure 1:**
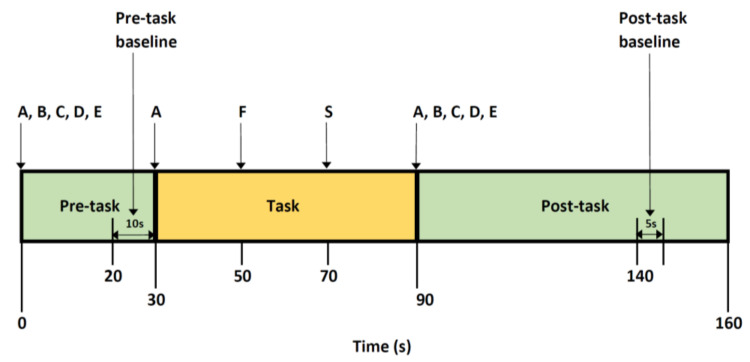
Verbal fluency test protocol.

The VFT assesses executive control and is commonly used to study cognitive activation in the frontotemporal cortices, as it involves an active search for specific information in the memory [54,55,56]. Participants were introduced to the task through a demonstration video and were given a short trial session to ensure they fully understood the assessment and were able to perform what was required of them during the actual trial. Subjects were instructed to avoid excessive head and body movements such as strong biting and yawning to prevent motion artefacts or modify cerebral perfusion for reasons unrelated to the task. They were instructed to remain seated upright and focus on the fixation cross displayed on the monitor with their eyes open throughout the measurements. The VFT paradigm used in previous studies was modified for the English language (Figure 1). It included a 30 seconds (s) pre-task period, a 60 s task period, and a 70 s post-task period [57]. The rate of sampling was 0.1 s. Subjects were directed to repeatedly enunciate a train of English letters “A, B, C, D, E” aloud in the pre- and post-task baseline periods. During the task period, they were instructed to generate as many English words beginning with the designated letter as possible. Subjects were given an auditory cue at the start and end of the task period and when the letters were changed. Task performance during the measurement period was assessed by the total number of correct and unique words generated during the 60 s task period.

### 2.3. NIRS Measurement and Signal Analysis

The current study used a continuous-wave NIRS system (ETG-4000, Hitachi Medical Co., Tokyo, Japan) with 52 channels, which detects cerebral blood flow by measuring relative changes in oxy-Hb and deoxy-Hb using two wavelengths (695 and 830 nm) of near-infrared light based on the modified Beer–Lambert law [58]. Each light source is paired with detectors arranged 3 cm apart, and each source–detector pair was able to measure 2–2.5 cm beneath the scalp surface [59,60]. The area between each pair was defined as a channel. The Cz location was positioned halfway along the distance between the nasion and the inion, and halfway along the distance between the pre-auricular points. The lowest probes were positioned along the Fp1–Fp2 line (a few centimeters above the eyebrows) based on the international 10–20 system used in electroencephalograms. This arrangement allowed for the quantification of haemoglobin changes in the prefrontal region, the frontopolar cortex, and the bilateral superior temporal cortical surface regions [61]. The 52-multi-channel NIRS system was able to detect multiple regions of interests based on the individual channels accounting for the respective anatomical areas of the brain are referenced in accordance with previous studies [62,63,64].

**Figure 2:**
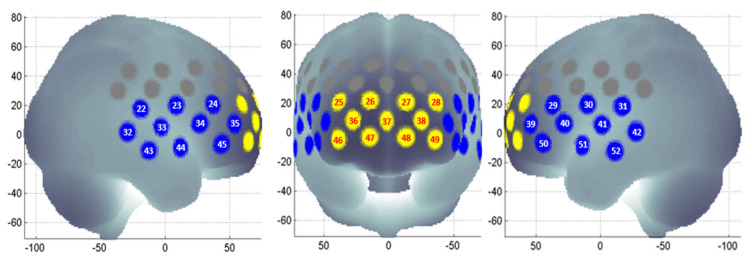
Regions of interest. Probe locations in the frontal (yellow) and temporal regions (blue) used for near-infrared spectroscopy (NIRS). Channel positions were plotted using the NFRI functions toolbox [67].

NIRS signals were processed according to the method described by Takizawa et al. [57]. Oxy-Hb, deoxy-Hb, and total-Hb concentrations were derived from optical densities using the modified Beer–Lambert law. Normalisation of the data was done through linear fitting in a 10 s baseline period before the task period, and the post-task baseline was fixed as the 50 s after the task period, with the mean 5 s value taken (Figure 1). Short-term motion artefacts were removed by applying a moving average factor of 5 s. Channels with artefacts, identified using an algorithm that automatically rejects channels influenced by body movements or fluctuations in frequency noise, were excluded from further analysis. In the event that a channel did not show good intensity due to poor contact between the optodes and the scalp (i.e., hair artefacts, poor angle), the optodes were first removed from the NIRSCap and the hair was pushed away from the contact zone. Following that, the optode was reattached to the NIRSCap in a perpendicular position. The process was repeated until nearly all optodes received an acceptable light intensity displayed on the data collection software interface. NIRS signals were analysed in clusters of channels in addition to a single-channel analysis to increase the robustness and reliability of the results [65]. Two clusters were identified: the frontal (11 channels, yellow) and temporal (20 channels, blue) regions (Figure 2). Subjects with less than six channels in either region of interest after software filtering were excluded from analysis. The haemodynamic response was quantified using two visual indices, namely integral value (magnitude of NIRS signal area during task period quantified using the haemodynamic response of oxy-Hb and averaging the signals from the channels in each region) and centroid value (midpoint time of the NIRS signal area throughout the entire task, inclusive of pre-task baseline, task period, and post-task baseline) [65,66].

### 2.4. Blood Collection and Metabolite Analysis

The serum metabolomic profiling analysis was performed at the Duke-NUS Metabolomics Facility, as described previously [68]. Serum samples were collected using vacutainer serum tubes (Becton & Dickinson, Singapore) and processed within one hour of collection. Samples were centrifuged for 10 min at 4 °C, at 2000× *g,* after clot formation to obtain the serum samples, which were stored at −80 °C and shipped on dry ice to the analytical laboratory. The researchers of the laboratory were blinded to the sample identification codes.

Serum samples of 50 ul were extracted using methanol and dried under nitrogen gas. The dried extracts were derivatised with 3 M of hydrochloric acid in butanol (Sigma Aldrich, St. Louis, MO, USA) and diluted in water for analysis in LC-MS. Deuterated stable isotopes of the respective amino acids were used as internal standards. A targeted analysis of amino acids was performed by liquid chromatography-mass spectrometry (LC-MS). LC-MS analysis were conducted on an Agilent 1290 Infinity LC system (Agilent Technologies, Santa Clara, CA, USA) coupled with a quadrupole-ion trap mass spectrometer (QTRAP 5500, AB Sciex, Olympia, DC, USA). The samples were separated using a C18 column (Phenomenex, 100 × 2.1 mm, 1.6 μm, Luna^®^ Omega, Torrance, CA, USA). Mobile phase A (Water) and Mobile phase B (Acetonitrile), both containing 0.1% formic acid, were used for chromatography separation. The LC run was performed at a flow rate of 0.4 mL min^−1^ with an initial gradient of 2% B for 0.8 min, followed by increases to 15% B in 0.1 min, 20% B in 5.7 min, 50% B in 0.5 min, and 70% B in 0.5 min, followed by re-equilibration of the column to the initial run conditions (2% B) for 0.9 min. All compounds were ionized in positive mode using electrospray ionization. The chromatograms were integrated using MultiQuant™ 3.0.3 software (AB Sciex, Olympia, DC, USA). Absolute quantification of amino acids was done by comparing the ratios of the metabolites with their respective internal standards, against an external calibration curve consisting of all reported amino acids. 

### 2.5. Statistical Analysis

All statistical analyses were performed using IBM SPSS Statistics 20.0. Demographic and clinical variables were compared between groups using the independent *t*-test and the chi-square test for continuous and categorical variables, respectively. Continuous variables included age, number of years of education, VFT task performance, integral value, centroid value, and HAM-D score. Categorical variables consisted of gender, ethnicity, handedness, and family history. Correlation analysis between haemodynamic responses and clinical characteristics were performed using Spearman’s correlation and Pearson’s correlation for categorical and continuous variables, respectively.

Activation of each channel during the task was determined using paired *t*-tests by comparing the changes in oxy-Hb and deoxy-Hb concentrations between the pre-task and task periods for each group. Independent *t*-tests were used to determine the effect of belonging to the diagnostic group on the haemodynamic response at each channel. A false discovery rate of 0.05 (two-tailed) was applied to limit false positive results to 5% [69]. Activation at the frontotemporal cortices and amino acid concentration were analysed by an analysis of covariance (ANCOVA) with the integral values, centroid values, and amino acid concentration used as dependent variables and diagnostic groups used as independent variables. Age, gender, and ethnicity were included as covariates to control for confounding. A post-hoc test with Fisher’s least significant difference (LSD) was used to evaluate significant differences between groups.

The classic receiver operating characteristic (ROC) analysis was used to evaluate the predictive performance of the integral and/or centroid values of the frontal and temporal regions and amino acid concentrations to classify HCs and MDD patients. A multivariable ROC analysis was done by coupling haemodynamic responses and amino acids concentrations. The likelihood test was used to plot the ROC curve, and the optimal cut-off point of the predicted probability was obtained by maximising Youden’s Index, which is a measure of the maximum potential effectiveness of a biomarker by balancing sensitivity and specificity. The effect size for statistically significant differences between groups was calculated using Hedge’s g. All tests were two-tailed, and a significance level of *p* ≤ 0.05 was used.

## 3. Results

### 3.1. Subject Demographics and Clinical Data

HCs and patients did not differ in any of the demographic variables studied (*p* > 0.05, Table 1). As compared with HCs, patients performed worse in the VFT (F1,44 = 4.769, *p* = 0.034; g = −0.603, 95% CI from −1.176 to −0.031) and had markedly higher HAM-D scores (F1,45 = 436.541, *p* < 0.001; g = 6.000, 95% CI 4.7–7.3). Approximately 60% of patients reported no prior admission to psychiatric wards or previous suicide attempts, and about 30% of patients were medication naïve. Patients receiving pharmacotherapy were on antidepressants, and a fraction were on combination anxiolytics, antipsychotics, or mood stabilisers. Details of the medications used by patients were highlighted in Table S2. On average, patients had been diagnosed with MDD for nearly a decade, and about 90% of patients had experienced recurrent depression. Correlation analysis found a negative relationship between the mean oxy-Hb levels in the OFC and the duration of depression (ρ = −0.465, *p* = 0.029), and did not identify any association between mean oxy-Hb levels and other clinical characteristics (Table S3).

### 3.2. Haemodynamic Response during the VFT

**Figure 3:**
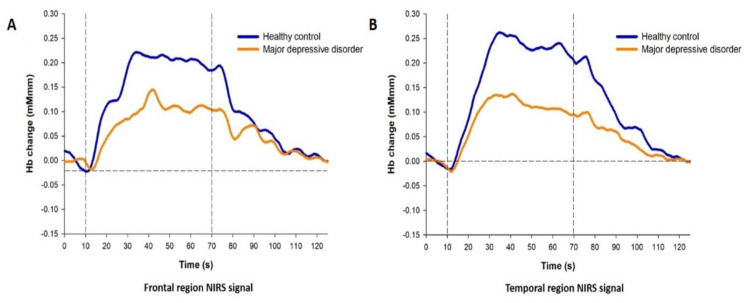
Average oxy-haemmoglobin waveforms in the (**A**) frontal and (**B**) temporal regions.

As compared with HCs, MDD patients had lower integral values in both the frontal (F1,41 = 6.741, *p* = 0.013; g = −0.771, 95% CI from −1.371 to −0.172) and temporal (F1,39 = 5.900, *p* = 0.020; g = −0.73, 95% CI from −1.341 to −0.12) regions (Figure 3). Centroid values did not differ between groups (frontal region, *p* = 0.155; temporal region, *p* = 0.185).

Among the 52 channels, 41 channels displayed marked increases in the mean oxy-Hb concentration during the task period relative to the pre-task baseline for HCs (*p*-values ≤ 0.001 to 0.038, Figure S1), and a simultaneous decrease in the mean deoxy-Hb concentration was observed in 23 channels (*p*-values ≤ 0.001 to 0.044, Figure S2). In HCs, activation occurred primarily in the frontal, temporal, and parietal lobes during the task period. In contrast, during the task, significant activation was observed in only 22 channels for mean oxy-Hb (*p*-values ≤ 0.001 to 0.047, Figure S1) and in nine channels for mean deoxy-Hb (*p*-values ≤ 0.001 to 0.046, Figure S2) for MDD patients. Activation was limited to the bilateral inferior frontal gyrus, left and right superior and middle frontal gyrus, right superior temporal gyrus, and the orbitofrontal area of MDD patients. The increases in oxy-Hb from baseline in these regions were much smaller in patients as compared with HCs (Figure S1).

In addition, when compared between groups, HCs had higher mean oxy-Hb concentrations than patients during task period in nine other channels located in the right and left postcentral gyrus, right supramarginal gyrus, and in the left superior frontal gyrus and inferior frontal gyrus (*p*-values from 0.004 to 0.034, Figure S3). At the same time, HCs had lower mean deoxy-Hb concentrations during task periods as compared with patients in 4 channels located in the OFC, supramarginal gyrus, and middle temporal gyrus (*p*-values from 0.013 to 0.042, Figure S4). Furthermore, patients had lower haemodynamic responses at Channel 38 (*p* = 0.005). Channel 38 is located in the left superior frontal gyrus, which corresponds to Brodmann’s area 10, known as the OFC [64].

**Figure 4:**
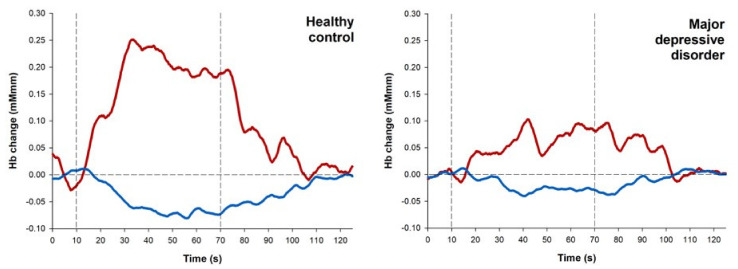
Mean oxy- and deoxy-Hb waveforms at Channel 38. Vertical dotted lines indicate the start and end of the task period.

Furthermore, the magnitude of the oxy-Hb response in HCs increased rapidly at the beginning of the task, remained at a peak level during the task, and gradually decreased in the post-task period in Channel 38. On the contrary, oxy-Hb levels in patients displayed a gradual and much lower increase during the task period and had slower decrease after the end of the task (Figure 4). These differential patterns are similar to previous findings [57,70]. These findings suggest that haemodynamic dysfunction occurs in the OFC of patients with MDD during the VFT, and the decline in haemodynamic response may be associated with the duration of depression.

### 3.3. Metabolite Analysis

The comparison of amino acid concentrations in HC and MDD patients showed a significantly higher concentration of histidine in HCs as compared with patients with MDD (F1,45 = 8.09, *p* = 0.007; d = −0.816, 95% CI from −1.431 to −0.201) (Table S1). Serum histidine levels were not associated with demographic and clinical factors for both HCs and MDD patients, and did not correlate with the severity of depression for MDD patients.

### 3.4. Differentiating MDD Patients from HCs

The area under the curve (AUC) for the frontal and temporal region integral values was 0.676 (95% CI 0.52–0.83) and 0.721 (95% CI 0.56–0.88), respectively. Using a threshold value of 80, the frontal region correctly classified 59.1% of patients (proportion of patients/measurements 13/22) and 62.5% of HCs (proportion of controls/measurements 15/24, positive predictive value (PPV) = 0.59, negative predictive value (NPV) = 0.63). Similarly, using an integral value of 86 at the temporal region correctly classified 59.1% (13/22) of patients and 63.6% of HCs (14/22, PPV = 0.62, NPV = 0.61). Likewise, the AUC for histidine was 0.712 (95% CI 0.57–0.86). Using an optimal value of 101 uM, serum histidine correctly classified 76.0% of patients (19/25) and 60.0% of HCs (15/25, PPV = 0.66, NPV = 0.71). The figure references can be found in the supplementary material (Figure S5).

**Figure 5:**
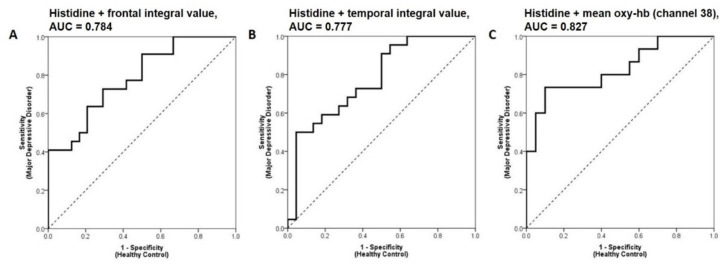
Receiver operating characteristic analyses using combined biomarkers of: (**A**) Frontal region integral values and histidine; (**B**) temporal region integral values and histidine; (**C**) histidine and the mean oxy-haemoglobin concentration in Channel 38 between patients with MDD and healthy controls. The optimal cut point was maximised using Youden’s Index.

To improve the predictive accuracy, the histidine concentration was coupled with integral values measured by NIRS to perform a multivariable ROC analysis. There was an overall improvement in predictive accuracy when combination markers were used (Figure 5) as compared with using a single marker (Figure S5). The AUC for histidine and the integral value of the frontal region increased to 0.784 (95% CI 0.65–0.91), and a predicted probability of 0.42 correctly classified 72.7% of patients (16/22) and 70.8% of HCs (17/24, PPV = 0.70, NPV = 0.74). Similarly, the AUC for histidine and the integral value of the temporal region improved to 0.777 (95% CI 0.64–0.91), and a predicted probability of 0.72 correctly classified 50.0% of patients (11/22) and 95.5% of controls (21/22, PPV = 0.92, NPV = 0.66). Moreover, the AUC for histidine combined with the mean oxy-Hb level at Channel 38 increased to 0.827 (95% CI 0.68–0.97), and a predicted probability of 0.50 correctly classified 73.3% of patients (11/15) and 90.0% of controls (18/20, PPV = 0.85, NPV = 0.82) (Figure 5).

## 4. Discussion

The present study reinforces the feasibility of utilising neuroimaging and metabolites as biosignatures for MDD diagnosis. fNIRS has promise for use as a non-invasive clinical measurement tool using infrared light by evaluating haemodynamic changes in the prefrontal cortical surface area. Consistent with previous findings, patients exhibited significantly reduced activation in the frontal and temporal lobes, regions known to be affected in MDD [18,71,72]. Lower activation in the frontotemporal cortices may suggest a regression in physiological function. fNIRS and fMRI studies found that MDD patients in remission exhibited reduced cerebral responses and functional abnormalities specifically in the left PFC, suggesting that dysfunction in the frontal lobe may be a specific trait marker for depression [58,73]. Fu et al. studied MDD patients using PET and reported diminished cerebral blood flow and glucose metabolism in the prefrontal lobes as well as attenuated glucose uptake in the frontal and temporal lobes [74,75]. Likewise, a single-photon emission computerized tomography (SPECT) study found lower perfusion in the PFC in patients with depression and established an association between hypofrontality in the frontotemporal cortices and a poor response to treatment in patients with depression [76,77]. Notably, disruptions in synaptic plasticity induced by depression have been correlated with volumetric changes in the PFC due to neuronal and glia atrophy [78] and memory impairment amongst patients with depression [79]. Although not specific to MDD, low frontotemporal activation is associated with the DSM-5 criteria for MDD [23]. Understanding that patients with MDD may display attenuated perfusion in the frontotemporal cortices may endow clinicians with better understanding of the underlying pathophysiology of their patients’ current state of disorders and help to explain cognitive impairments or behavioural problems, and therefore improve the effectiveness of treatments and avoid long-term complications for patients.

Another significant finding in this study was the negative association between the duration of depression and the mean oxy-Hb level in the left OFC. The duration of depression has a substantial impact on the disease prognosis and treatment response and may provide clues about the depression severity and the likelihood of improvement with future treatment [80], especially because the OFC is crucial in decision-making functions and linked to subcortical areas related to memory, learning and attention [81]. Patients with a longer illness duration are likely to experience chronic stress and have a greater risk of recurrence within five years [82,83]. In addition, from an immunological perspective, patients may have increased microglial activation, which is an indicator of neuropathological progression [84]. Microglial activation in response to stress in depression has been implicated in a dysfunctional hypothalamus–pituitary–adrenal (HPA) axis and higher cortisol levels [85]. The disparity of inflammation in the CNS promotes the release of cytokines and upsets the functional coupling and structural changes, leading to impaired neurovascular responsivity and reduced cerebral blood flow following neural stimulation [86]. In addition to attenuated brain activity, structural deficits support the findings from functional imaging that there are volumetric reductions in grey matter [87], hypometabolism during the resting state [88], increased vulnerability to cellular apoptosis [89], and altered connectivity [90] in the OFC; Tsujii et al. proposed that the OFC is a potential area that could be used to discern between MDD and bipolar disorder, two disorders with similar symptoms [91]. Moreover, the OFC may be a promising stimulation target for the treatment of MDD. Preliminary evidence found improvements in mood among individuals with moderate to severe depression when electrical stimulation was applied to the lateral ends of the OFC [92]. The importance of the OFC as a potential site for differential diagnosis of psychiatric disorders and treatment response is in line with our study, which highlighted Channel 38 (corresponding to the OFC region) as a potential biomarker that can differentiate depressed from non-depressed individuals and is correlated with the duration of depression.

Cross-sectional studies have explored the potential of amino acids to be used for the development of specific diagnostic tools. However, mixed results have left the issue of whether they can be used in clinical psychiatry as validated biomarkers or as a proxy indicator unresolved. The amino acid profiles of MDD patients did not show improvements despite treatment, although it is suggested that amino acids helped to alleviate the symptomatology of depression [24,93]. Similarly, Maes et al. proposed the concentrations of amino acids as being predictive of treatment responsivity, despite there being no significant differences between MDD patients and healthy subjects [94]. In contrast, Xu et al. reported its clinical utility in diagnosing MDD [46]. More recently, a biomarker panel demonstrated the feasibility of differentiating two commonly misdiagnosed psychiatric disorders, MDD and bipolar disorder, with a reliability of nearly 90% [44]. Furthermore, the use of a long-term appropriate treatment regimen could likely restore the blood concentrations of amino acids [95]. Thus, alterations in amino acid metabolism and compounds seem to play important roles in the etiopathogenesis of psychiatric disorders, which may involve circulation–brain transport dysfunction or altered capacity of enzymes in the metabolic pathway.

Among the 17 metabolites analysed, only histidine was found to be significantly lower in patients. Histidine is an essential amino acid that is used for the biosynthesis of proteins and is the precursor for several hormones, including the noteworthy neurotransmitter histamine. Unsurprisingly, histidine deficiency has a strong relationship with common depressive symptoms, such as anxiety, mental fatigue, sleep disruption, inattention, and cognitive impairment, and it appears to be altered in the circulation of patients with depressive disorders [96,97,98,99]. Histidine deficiencies in MDD patients can be accounted for by the chronic catabolic status present in MDD patients caused by a loss of appetite [100]. Circulating histidine is transported through the BBB by a carrier-mediated transport system by altering the cellular permeability [101,102] and converted to histamine in the brain. However, disruption of the regulatory mechanisms of the CNS which is often found in depressed patients can hinder this process. The process is dependent on the rate-limiting enzyme histidine decarboxylase and is regulated by the TCF4 gene. TCF4 has been implicated in many common CNS disorders due to its control of the neurodevelopmental pathway [103]. Patients with recurrent depression have lower levels of TCF4 expression, and this, in turn, accelerates the decarboxylation process of histidine by releasing its inhibition on histidine decarboxylase, promoting histamine synthesis [25,104,105,106].

The modulation of physiological functions and behaviours by the histaminergic system is disrupted under stressful conditions. In the brain, histamine is exclusively synthesised by histaminergic neurons located at the hypothalamic tuberomammillary nuclei, and it is primarily involved in the regulation of wakefulness, sleep, motivation, and cognition [107,108,109,110]. In particular, changes in the density of H1 and H3 receptors are associated with the development of mood disorders due to their involvement in the stress response and neurotransmitter regulation [111,112]. Endogenous histamine is elevated alongside lower neuronal H3 receptors in the mammalian brain under conditions of acute stress [113,114], and chronic release of histamine under repeated stress may result in the downregulation of H1 receptors [115,116,117]. Interestingly, the depletion of H1 receptors in depressed patients is positively correlated with the severity of clinical symptoms [118,119]. Reduced receptor density and increased activity of the histamine metabolising enzyme may trigger a compensatory mechanism with high histamine turnover, leading to histamine intolerance and increased vulnerability to stress-induced depression [120]. Furthermore, disruption of the histaminergic neuronal system conversely disturbs the serotonergic system, as the two systems have demonstrated shared control of impaired cognitive functioning in depression [121,122]. Although the aetiology of depression remains poorly understood, it is likely to stem from the interaction between genetic and environmental factors, with heritability studies indicating a two- to three-fold increased risk of developing MDD in genetically susceptible individuals [123,124,125].

With current scientific advances, the discovery of novel markers for use in early detection and prognostication in MDD is highly anticipated. However, the nature of depressive disorders and their high heterogenicity make it difficult to identify a single biomarker that satisfies both high sensitivity and specificity requirements for the disease. To the bes of our knowledge, this is the first study to evaluate the applicability of combining markers using the fNIRS paradigm and amino acid data to discriminate MDD patients from healthy subjects with improved levels of sensitivity and specificity. The regions of interests (integral values at the frontal and temporal lobe) displayed much lower sensitivity and specificity in distinguishing MDD patients from HC using a single marker. The results of our multivariable ROC analyses revealed that cortical activation magnitudes, when integrated with amino acid profiles, were able to distinguish between MDD patients and HCs with significantly higher accuracy. Combination markers were shown to perform better than single-marker diagnostic tests, and the incremental usefulness of adding multiple markers for disease prediction encourages the identification of additional markers to enhance the specificity of conventional tests. Hence, biomarker panels and diagnostic tests that combine marker values are now the focus of the research and clinical fields alike. Still, the favourable prospects of combining biomarkers for clinical diagnosis is challenged by the lack of a standardised methodology to quantify the diagnostic gains, despite an array of clinical literature [126]. Although predicted probabilities are unlikely to be arbitrary, it is still unclear how combination rules have been developed or whether the improvements are sufficiently robust against varying study designs and research populations. Nonetheless, the discovery of possible signatures to improve the clinical reliability in MDD diagnosis is anticipated and very much needed as a steppingstone for the advancement of precision medicine in psychiatry.

This study has several limitations. Restricted by depth sensitivity, NIRS is unable to detect deep brain areas such as the hippocampus and thalamus, and the lack of transmission through the cerebral cortex may underestimate haemoglobin changes. Overlapping measurements using fMRI or PET can alleviate this concern, as fiber optic cables do not interfere with the measurements, hence, enabling multimodal studies as demonstrated by Strangman et al. [127]. Furthermore, Shin et al. and Zama et al. demonstrated that such multimodal integration was able to supplement single modality’s limitations and yielded improved and reliable results by utilising complementary features [128,129]. Nevertheless, such studies and data are currently limited and more needs to be done to investigate the feasibility of simultaneous measurements. Another challenge is that NIRS measurements are relative to the baseline, and findings may be due to a hyperperfusion during the pre-task period in patients. However, MRI studies have reported hypofusion in the frontoparietal areas of MDD patients at rest [130]. Thus, decreased activation in patients is unlikely to be due to a saturated hemodynamic state in the pre-task period. Ideally, metabolite samples should be collected in the morning, and dietary restrictions are required on the day prior. Furthermore, the metabolite analysis should not be limited to just a single metabolite (in this case, amino acids) to allow for better identification of target markers. However, this study aims to identify a fast and effective diagnostic methodology that is able to quickly identify and distinguish MDD patients with a stable medical condition from healthy individuals to be able to administer the necessary treatment for higher effectiveness. Although the lack of significant findings for other amino acids may potentially be due to treatment effects, as not all MDD patients are medication-naïve [25,106], restricting diets and testing multiple metabolites is not clinically feasible in this study, with the limited time available and doing so could further deter patients from consenting to treatment due to higher costs and delayed treatment. Studies have reported that the time of day for blood collection or imposing blood collection requirements such as fasting did not influence the variability in amino acid concentrations within a short time span (48 h) prior to blood collection [131]. The cross-sectional nature of the study renders it impossible to establish a cause-and-effect relationship between the onset of depression and haemodynamic responses. The lack of associations between haemodynamic responses and clinical factors may have been liable to the small sample size, and also deterred the possibility of identifying the relationship between cortical activation and clinical symptoms of depression in different subtypes of MDD. However, combined biomarkers may warrant more accurate results with fewer subjects with greater diagnostic performance. Despite its limitations, this study provides new evidence on the use of combination markers to better diagnose MDD patients using two established biological methodologies, and it contributes to the existing limited body of literature on precision psychiatry. Further studies involving larger populations are warranted to determine the utility of these biomarkers in clinical practice.

## 5. Conclusions

In summary, our study found that in integrating cortical activity magnitudes with amino acid profiles, a multivariable ROC curve was able to distinguish between patients with MDD and HCs with improved sensitivity and specificity, hence, demonstrating the viability of combination biomarkers for use in MDD diagnosis. The diminished haemodynamic response in the cortices of MDD patients and reduced metabolite concentrations further lend support for the investigation of haemodynamic activities, coupled with homeostatic responses, underlying the biological basis of the disorder. The ultimate goal of improving disease treatment in patients with MDD to improve their quality of life can be achieved, as precision psychiatry may reduce the translational gap in the development of diagnostic tools capable of refining diagnosis, ascertaining prognosis, guiding treatment, and predicting treatment responses. NIRS values and amino acid concentrations can be used to provide an objective assessment of MDD patients with depressive symptoms and may be used to develop candidate biosignatures that allow clinicians to improve the inter-rater reliability of psychiatric diagnosis. Our findings also form the groundwork for future research investigating biomarker combinations for MDD with optimal diagnostic and prediction performance.

## Figures and Tables

**Table 1:** Demographic and clinical characteristics.

	MDD (*n* = 25)	HC (*n* = 25)	*p*-Value
Age (years)	29.5 ± 8.7	30.3 ± 8.6	0.744
Gender			1.000
Male	7 (28.0%)	7 (28.0%)	
Female	18 (72.0%)	18 (72.0%)	
Ethnicity			1.000
Chinese	18 (72.0%)	18 (72.0%)	
Malay	3 (12.0%)	3 (12.0%)	
Indian	4 (16.0%)	4 (16.0%)	
Handedness			0.307
Right	22 (84.0%)	24 (96.0%)	
Ambidextrous	3 (12.0%)	1 (4.0%)	
Education (years)	13.8 ± 2.0	14.2 ± 1.9	0.518
VFT task performance ^a^	19.4 ± 4.6	22.3 ± 5.2	**0.034**
HAM-D score	21.5 ± 4.3	1.9 ± 1.8	**≤0.001**
Family psychiatric history	11 (44.0%)	6 (24.0%)	0.136
Age at onset (years)	20.9 ± 6.4	-	
Duration of illness (years)	9.1 ± 8.3	-	
Duration of untreated illness (months)	58.1 ± 65.7	-	
Past admission to psychiatric ward	9 (36.0%)	-	
Past suicide attempts	9 (36.0%)	-	
Episode			
Single	2 (8.0%)	-	
Recurrent	23 (92.0%)	-	
Depression severity			
Mild	4 (16.0%)	-	
Moderate	13 (52.0%)	-	
Severe	8 (32.0%)	-	
Pharmacotherapy	17 (68.0%)	-	
Fluoxetine equivalent dose (mg/day)	44.3 ± 28.8	-	
Diazepam equivalent dose (mg/day)	3.1 ± 1.4	-	
Chlorpromazine equivalent dose (mg/day)	125.5 ± 133.3	-	

Mean ± SD are shown and *p*-values ≤0.05 are in bold. Depression severity was determined based on the Hamilton Depression Rating Scale. ^a^ Complete demographic data was not obtained for all subjects. (Known verbal fluency test (VFT) task performance in major depressive disorder (MDD) patients, *n* = 24). HC, healthy control.

## Data Availability

The data presented in this study are available on request from the corresponding author.

## Supplementary Materials

The following are available online at https://www.mdpi.com/article/10.3390/diagnostics11111978/s1, Table S1: Measurements of amino acids in 25 healthy controls and 25 major depressive disorder subjects, Table S2: Medication details, Table S3: Correlations between integral values and clinical characteristics in major depressive disorder, Figure S1: Activation at each channel determined by paired sample *t*-test comparing the mean oxy-haemoglobin levels during pre-task baseline period and task period, Figure S2: Activation at each channel determined by paired sample *t*-test comparing the mean deoxy-haemoglobin levels during pre-task baseline period and task period, Figure S3: Group differences in mean oxy-haemoglobin levels during task period determined by Student’s *t*-test, Figure S4: Group differences in mean deoxy-haemoglobin levels during task period determined by Student’s *t*-test, Figure S5: Receiver operating characteristic analysis of single biomarkers between MDD patients and healthy controls.

## References

[1] Pizzagalli D.A. (2014). Depression, Stress, and Anhedonia: Toward a Synthesis and Integrated Model. Annu. Rev. Clin. Psychol..

[2] Kessler R.C., Bromet E.J. (2013). The Epidemiology of Depression Across Cultures. Annu. Rev. Public Health.

[3] McIntyre R.S., Cha D.S., Soczynska J., Woldeyohannes H.O., Gallaugher L.A., Kudlow P., Alsuwaidan M., Baskaran A. (2013). Cognitive deficits and functional outcomes in major depressive disorder: Determinants, substrates, and treatment interventions. Depress. Anxiety.

[4] Rock P.L., Roiser J., Riedel W., Blackwell A.D. (2013). Cognitive impairment in depression: A systematic review and meta-analysis. Psychol. Med..

[5] Smith K. (2014). Mental health: A world of depression. Nature.

[6] Mathers C.D., Loncar D. (2006). Projections of Global Mortality and Burden of Disease from 2002 to 2030. PLoS Med..

[7] Cui R. (2015). Editorial: A Systematic Review of Depression. Curr. Neuropharmacol..

[8] Luo Y., Zhang S., Zheng R., Xu L., Wu J. (2018). Effects of depression on heart rate variability in elderly patients with stable coronary artery disease. J. Evid.-Based Med..

[9] Bilello J.A. (2016). Seeking an objective diagnosis of depression. Biomark. Med..

[10] Hacimusalar Y., Esel E. (2017). Suggested Biomarkers for Major Depressive Disorder. Arch. Neuropsychiatry.

[11] Gur R.E., Keshavan M.S., Lawrie S.M. (2007). Deconstructing Psychosis With Human Brain Imaging. Schizophr. Bull..

[12] Cynthia Y., Lai Y., Charmaine C.S.H.H., Lim R., Roger C., Ho M. (2017). Functional near-infrared spectroscopy in psychiatry. BJPsych Adv..

[13] Gsell W., De Sadeleer C., Marchalant Y., MacKenzie E.T., Schumann P., Dauphin F. (2000). The use of cerebral blood flow as an index of neuronal activity in functional neuroimaging: Experimental and pathophysiological considerations. J. Chem. Neuroanat..

[14] Scholkmann F., Kleiser S., Metz A.J., Zimmermann R., Pavia J.M., Wolf U., Wolf M. (2014). A review on continuous wave functional near-infrared spectroscopy and imaging instrumentation and methodology. Neuroimage.

[15] Ferrari M., Quaresima V. (2012). A brief review on the history of human functional near-infrared spectroscopy (fNIRS) development and fields of application. NeuroImage.

[16] Szameitat A.J., Shen S., Sterr A. (2009). The functional magnetic resonance imaging (fMRI) procedure as experienced by healthy participants and stroke patients—A pilot study. BMC Med. Imaging.

[17] Huang B., Law M.W.-M., Khong P.-L. (2009). Whole-Body PET/CT Scanning: Estimation of Radiation Dose and Cancer Risk. Radiology.

[18] Husain S.F., Yu R., Tang T.-B., Tam W.W., Tran B., Quek T.T., Hwang S.-H., Chang C.W., Ho C.S., Ho R.C. (2020). Validating a functional near-infrared spectroscopy diagnostic paradigm for Major Depressive Disorder. Sci. Rep..

[19] Husain S.F., Tang T.-B., Yu R., Tam W.W., Tran B., Quek T.T., Hwang S.-H., Chang C.W., Ho C.S., Ho R.C. (2019). Cortical haemodynamic response measured by functional near infrared spectroscopy during a verbal fluency task in patients with major depression and borderline personality disorder. EBioMedicine.

[20] Almeida J., Versace A., Mechelli A., Hassel S., Quevedo K., Kupfer D.J., Phillips M.L. (2009). Abnormal Amygdala-Prefrontal Effective Connectivity to Happy Faces Differentiates Bipolar from Major Depression. Biol. Psychiatry.

[21] De Oliveira G.N., Kummer A., Salgado J.V., Portela E.J., Sousa-pereira S.R., David A.S., Teixeira A.L. (2010). Psychiatric disorders in temporal lobe epilepsy: An overview from a tertiary service in Brazil. Seizure.

[22] Mulder D.W., Daly D. (1952). Psychiatric symptoms associated with lesions of temporal lobe. J. Am. Med. Assoc..

[23] Zhang H., Dong W., Dang W., Quan W., Tian J., Chen R., Zhan S., Yu X. (2014). Near-infrared spectroscopy for examination of prefrontal activation during cognitive tasks in patients with major depressive disorder: A meta-analysis of observational studies. Psychiatry Clin. Neurosci..

[24] Altamura C., Maes M., Dai J., Meltzer H. (1995). Plasma concentrations of excitatory amino acids, serine, glycine, taurine and histidine in major depression. Eur. Neuropsychopharmacol..

[25] Kofler M., Schiefecker A.J., Gaasch M., Sperner-Unterweger B., Fuchs D., Beer R., Ferger B., Rass V., Hackl W., Rhomberg P. (2019). A reduced concentration of brain interstitial amino acids is associated with depression in subarachnoid hemorrhage patients. Sci. Rep..

[26] Demyer M.K., Shea P.A., Hendrie H.C., Yoshimura N.N. (1981). Plasma Tryptophan and Five Other Amino Acids in Depressed and Normal Subjects. Arch. Gen. Psychiatry.

[27] Moreira F.P., Jansen K., Cardoso T.D.A., Mondin T.C., Magalhães P.V., Kapczinski F., Souza L.D., Da Silva R.A., Oses J.P., Wiener C.D. (2019). Metabolic syndrome and psychiatric disorders: A population-based study. Rev. Bras. Psiquiatr..

[28] Dunbar J.A., Reddy P., Davis-Lameloise N., Philpot B., Laatikainen T., Kilkkinen A., Bunker S., Best J., Vartiainen E., Lo S.K. (2008). Depression: An Important Comorbidity With Metabolic Syndrome in a General Population. Diabetes Care.

[29] Brown G.L., Ebert M.H., Goyer P.F., Jimerson D.C., Klein W.J., Bunney W.E., Goodwin F.K. (1982). Aggression, suicide, and serotonin: Relationships to CSF amine metabolites. Am. J. Psychiatry.

[30] Diehl D.J., Gershon S. (1992). The role of dopamine in mood disorders. Compr. Psychiatry.

[31] Stockmeier C.A. (1997). Neurobiology of serotonin in depression and suicide. Ann. N. Y. Acad. Sci..

[32] Van Praag H.M. (1982). Depression, suicide and the metabolism of serotonin in the brain. J. Affect. Disord..

[33] Munari L., Provensi G., Passani M.B., Galeotti N., Cassano T., Benetti F., Corradetti R., Blandina P. (2015). Brain Histamine Is Crucial for Selective Serotonin Reuptake Inhibitors‘ Behavioral and Neurochemical Effects. Int. J. Neuropsychopharmacol..

[34] Airaksinen M.S., Flügge G., Fuchs E., Panula P. (1989). Histaminergic system in the tree shrew brain. J. Comp. Neurol..

[35] Laitinen K.S., Tuomisto L., Laitinen J.T. (1995). Endogenous serotonin modulates histamine release in the rat hypothalamus as measured by in vivo microdialysis. Eur. J. Pharmacol..

[36] Brown R.E., Sergeeva O.A., Eriksson K.S., Haas H.L. (2002). Convergent Excitation of Dorsal Raphe Serotonin Neurons by Multiple Arousal Systems (Orexin/Hypocretin, Histamine and Noradrenaline). J. Neurosci..

[37] Brigitta B. (2002). Pathophysiology of depression and mechanisms of treatment. Dialog. Clin. Neurosci..

[38] Anthony T.G. (2015). Homeostatic responses to amino acid insufficiency. Anim. Nutr..

[39] Firk C., Markus C.R. (2007). Review: Serotonin by stress interaction: A susceptibility factor for the development of depression?. J. Psychopharmacol..

[40] Leonard B.E. (1997). The role of noradrenaline in depression: A review. J. Psychopharmacol..

[41] McLean A., Rubinsztein J.S., Robbins T.W., Sahakian B.J. (2003). The effects of tyrosine depletion in normal healthy volunteers: Implications for unipolar depression. Psychopharmacology.

[42] Lakhan S.E., Vieira K.F. (2008). Nutritional therapies for mental disorders. Nutr. J..

[43] Lucca A., Lucini V., Catalano M., Smeraldi E. (1995). Neutral amino acid availability in two major psychiatric disorders. Prog. Neuro-Psychopharmacol. Biol. Psychiatry.

[44] Chen J.-J., Zhou C.-J., Liu Z., Fu Y.-Y., Zheng P., Yang D.-Y., Li Q., Mu J., Wei Y.-D., Zhou J.-J. (2015). Divergent Urinary Metabolic Phenotypes between Major Depressive Disorder and Bipolar Disorder Identified by a Combined GC–MS and NMR Spectroscopic Metabonomic Approach. J. Proteome Res..

[45] Islam R., Ali S., Karmoker J.R., Kadir M.F., Ahmed M.U., Nahar Z., Islam S.M.A., Islam M.S., Hasnat A., Islam S. (2020). Evaluation of serum amino acids and non-enzymatic antioxidants in drug-naïve first-episode major depressive disorder. BMC Psychiatry.

[46] Xu H.-B., Fang L., Hu Z.-C., Chen Y.-C., Chen J.-J., Li F.-F., Lu J., Mu J., Xie P. (2012). Potential clinical utility of plasma amino acid profiling in the detection of major depressive disorder. Psychiatry Res..

[47] Ahmed A.T., MahmoudianDehkordi S., Bhattacharyya S., Arnold M., Liu D., Neavin D., Moseley M.A., Thompson J.W., Williams L.S.J., Louie G. (2019). Acylcarnitine metabolomic profiles inform clinically-defined major depressive phenotypes. J. Affect. Disord..

[48] Dinoff A., Saleem M., Herrmann N., Mielke M.M., Oh P.I., Venkata S.L.V., Haughey N.J., Lanctôt K.L. (2017). Plasma sphingolipids and depressive symptoms in coronary artery disease. Brain Behav..

[49] Fernstrom J.D. (1981). Dietary Precursors and Brain Neurotransmitter Formation. Annu. Rev. Med..

[50] Inoshita M., Umehara H., Watanabe S.-Y., Nakataki M., Kinoshita M., Tomioka Y., Tajima A., Numata S., Ohmori T. (2018). Elevated peripheral blood glutamate levels in major depressive disorder. Neuropsychiatr. Dis. Treat..

[51] Schneider B., Prvulovic D. (2013). Novel biomarkers in major depression. Curr. Opin. Psychiatry.

[52] Breitenstein B., Scheuer S., Holsboer F. (2014). Are there meaningful biomarkers of treatment response for depression?. Drug Discov. Today.

[53] Zimmerman M., Martinez J.H., Young D., Chelminski I., Dalrymple K. (2013). Severity classification on the Hamilton depression rating scale. J. Affect. Disord..

[54] Herrmann M., Ehlis A.-C., Fallgatter A. (2003). Frontal activation during a verbal-fluency task as measured by near-infrared spectroscopy. Brain Res. Bull..

[55] Herrmann M., Walter A., Ehlis A.-C., Fallgatter A. (2006). Cerebral oxygenation changes in the prefrontal cortex: Effects of age and gender. Neurobiol. Aging.

[56] Fisk J.E., Sharp C.A. (2004). Age-Related Impairment in Executive Functioning: Updating, Inhibition, Shifting, and Access. J. Clin. Exp. Neuropsychol..

[57] Takizawa R., Kasai K., Kawakubo Y., Marumo K., Kawasaki S., Yamasue H., Fukuda M. (2008). Reduced frontopolar activation during verbal fluency task in schizophrenia: A multi-channel near-infrared spectroscopy study. Schizophr. Res..

[58] Tomioka H., Yamagata B., Kawasaki S., Pu S., Iwanami A., Hirano J., Nakagome K., Mimura M. (2015). A Longitudinal Functional Neuroimaging Study in Medication-Naïve Depression after Antidepressant Treatment. PLoS ONE.

[59] Okada E., Delpy D.T. (2003). Near-infrared light propagation in an adult head model I Modeling of low-level scattering in the cerebrospinal fluid layer. Appl. Opt..

[60] Okada E., Delpy D.T. (2003). Near-infrared light propagation in an adult head model II Effect of superficial tissue thickness on the sensitivity of the near-infrared spectroscopy signal. Appl. Opt..

[61] Chou P.H., Huang C.-J., Sun C.-W. (2020). The Potential Role of Functional Near-Infrared Spectroscopy as Clinical Biomarkers in Schizophrenia. Curr. Pharm. Des..

[62] Naganuma H., Tokumasu K., Hashimoto S., Okamoto M., Yamashina S. (2003). Three-dimensional analysis of morphological aspects of the human utricular macula. Ann. Otol. Rhinol. Laryngol..

[63] Tsuzuki D., Dan I. (2013). Spatial registration for functional near-infrared spectroscopy: From channel position on the scalp to cortical location in individual and group analyses. NeuroImage.

[64] Sato H., Yahata N., Funane T., Takizawa R., Katura T., Atsumori H., Nishimura Y., Kinoshita A., Kiguchi M., Koizumi H. (2013). A NIRS-fMRI investigation of prefrontal cortex activity during a working memory task. Neuroimage.

[65] Takizawa R., Fukuda M., Kawasaki S., Kasai K., Mimura M., Pu S., Noda T., Niwa S.-I., Okazaki Y. (2013). Neuroimaging-aided differential diagnosis of the depressive state. NeuroImage.

[66] Chou P.-H., Yao Y.-H., Zheng R.-X., Liou Y.-L., Liu T.-T., Lane H.-Y., Yang A.C., Wang S.-C. (2021). Deep Neural Network to Differentiate Brain Activity Between Patients With First-Episode Schizophrenia and Healthy Individuals: A Multi-Channel Near Infrared Spectroscopy Study. Front. Psychiatry.

[67] Jichi Medical University (2010). NIRS Tools. http://www.jichi.ac.jp/brainlab/tools.html.

[68] Kovalik J.-P., Zhao X., Gao F., Leng S., Chow V., Chew H., Teo L.L., Tan R.S., Ewe S.H., Tan H.C. (2021). Amino acid differences between diabetic older adults and non-diabetic older adults and their associations with cardiovascular function. J. Mol. Cell. Cardiol..

[69] Singh A.K., Dan I. (2006). Exploring the false discovery rate in multichannel NIRS. NeuroImage.

[70] Suto T., Fukuda M., Ito M., Uehara T., Mikuni M. (2004). Multichannel near-infrared spectroscopy in depression and schizophrenia: Cognitive brain activation study. Biol. Psychiatry.

[71] Qin J., Liu H., Wei M., Zhao K., Chen J., Zhu J., Shen X., Yan R., Yao Z., Lu Q. (2017). Reconfiguration of hub-level community structure in depressions: A follow-up study via diffusion tensor imaging. J. Affect. Disord..

[72] Halari R., Simic M., Pariante C.M., Papadopoulos A., Cleare A., Brammer M., Fombonne E., Rubia K. (2009). Reduced activation in lateral prefrontal cortex and anterior cingulate during attention and cognitive control functions in medication-naïve adolescents with depression compared to controls. J. Child Psychol. Psychiatry.

[73] Okada G., Okamoto Y., Yamashita H., Ueda K., Takami H., Yamawaki S. (2009). Attenuated prefrontal activation during a verbal fluency task in remitted major depression. Psychiatry Clin. Neurosci..

[74] Fu C., Shi D., Gao Y., Xu J. (2017). Functional assessment of prefrontal lobes in patients with major depression disorder using a dual-mode technique of 3D-arterial spin labeling and 18F-fluorodeoxyglucose positron emission tomography/computed tomography. Exp. Ther. Med..

[75] Fu C., Zhang H., Xuan A., Gao Y., Xu J., Shi D. (2018). A combined study of 18F-FDG PET-CT and fMRI for assessing resting cerebral function in patients with major depressive disorder. Exp. Ther. Med..

[76] Brockmann H., Zobel A., Joe A., Biermann K., Scheef L., Schuhmacher A., von Widdern O., Metten M., Biersack H.-J., Maier W. (2009). The value of HMPAO SPECT in predicting treatment response to citalopram in patients with major depression. Psychiatry Res. Neuroimaging.

[77] Amen D.G., Trujillo M., Newberg A., Willeumier K., Tarzwell R., Wu J.C., Chaitin B. (2011). Brain SPECT Imaging in Complex Psychiatric Cases: An Evidence-Based, Underutilized Tool. Open Neuroimaging J..

[78] Liu W., Ge T., Leng Y., Pan Z., Fan J., Yang W., Cui R. (2017). The Role of Neural Plasticity in Depression: From Hippocampus to Prefrontal Cortex. Neural Plast..

[79] Burt D.B., Zembar M.J., Niederehe G. (1995). Depression and memory impairment: A meta-analysis of the association, its pattern, and specificity. Psychol. Bull..

[80] Hung C.-I., Liu C.-Y., Yang C.-H. (2017). Untreated duration predicted the severity of depression at the two-year follow-up point. PLoS ONE.

[81] Kringelbach M.L., Rolls E.T. (2004). The functional neuroanatomy of the human orbitofrontal cortex: Evidence from neuroimaging and neuropsychology. Prog. Neurobiol..

[82] Burcusa S.L., Iacono W.G. (2007). Risk for recurrence in depression. Clin. Psychol. Rev..

[83] Shea M.T., Elkin I., Imber S.D., Sotsky S.M., Watkins J.T., Collins J.F., Pilkonis P.A., Beckham E., Glass D.R., Dolan R.T. (1992). Course of depressive symptoms over follow-up. Findings from the National Institute of Mental Health Treatment of Depression Collaborative Research Program. Arch Gen Psychiatry.

[84] Setiawan E., Attwells S., Wilson A.A., Mizrahi R., Rusjan P., Miler L., Xu C., Sharma S., Kish S., Houle S. (2018). Association of translocator protein total distribution volume with duration of untreated major depressive disorder: A cross-sectional study. Lancet Psychiatry.

[85] Lopez-Duran N.L., McGinnis E., Kuhlman K., Geiss E., Vargas I., Mayer S. (2015). HPA-axis stress reactivity in youth depression: Evidence of impaired regulatory processes in depressed boys. Stress.

[86] Elbau I.G., Brücklmeier B., Uhr M., Arloth J., Czamara D., Spoormaker V.I., Czisch M., Stephan K.E., Binder E.B., Sämann P.G. (2018). The brain’s hemodynamic response function rapidly changes under acute psychosocial stress in association with genetic and endocrine stress response markers. Proc. Natl. Acad. Sci. USA.

[87] Drevets W.C. (2007). Orbitofrontal Cortex Function and Structure in Depression. Ann. N. Y. Acad. Sci..

[88] Koob G., Volkow N. (2021). Neurocircuitry of Addiction. Neuropsychopharmacology.

[89] Miguel-Hidalgo J.J., Whittom A., Villarreal A., Soni M., Meshram A., Pickett J.C., Rajkowska G., Stockmeier C.A. (2014). Apoptosis-related proteins and proliferation markers in the orbitofrontal cortex in major depressive disorder. J. Affect. Disord..

[90] Helm K., Viol K., Weiger T.M., Tass P.A., Grefkes C., del Monte D., Schiepek G. (2018). Neuronal connectivity in major depressive disorder: A systematic review. Neuropsychiatr. Dis. Treat..

[91] Tsujii N., Otsuka I., Okazaki S., Yanagi M., Numata S., Yamaki N., Kawakubo Y., Shirakawa O., Hishimoto A. (2019). Mitochondrial DNA Copy Number Raises the Potential of Left Frontopolar Hemodynamic Response as a Diagnostic Marker for Distinguishing Bipolar Disorder From Major Depressive Disorder. Front. Psychiatry.

[92] Rao V.R., Sellers K.K., Wallace D.L., Lee M.B., Bijanzadeh M., Sani O.G., Yang Y., Shanechi M.M., Dawes H.E., Chang E.F. (2018). Direct Electrical Stimulation of Lateral Orbitofrontal Cortex Acutely Improves Mood in Individuals with Symptoms of Depression. Curr. Biol..

[93] Woo H.I., Chun M.-R., Yang J.-S., Lim S.-W., Kim M.-J., Kim S.-W., Myung W.-J., Kim D.-K., Lee S.-Y. (2015). Plasma amino acid profiling in major depressive disorder treated with selective serotonin reuptake inhibitors. CNS Neurosci. Ther..

[94] Maes M., Verkerk R., Vandoolaeghe E., Lin A., Scharpé S. (1998). Serum levels of excitatory amino acids, serine, glycine, histidine, threonine, taurine, alanine and arginine in treatment-resistant depression: Modulation by treatment with antidepressants and prediction of clinical responsivity. Acta Psychiatr. Scand..

[95] Schmidt H.D., Shelton R.C., Duman R.S. (2011). Functional Biomarkers of Depression: Diagnosis, Treatment, and Pathophysiology. Neuropsychopharmacology.

[96] Schön M., Mousa A., Berk M., Chia W.L., Ukropec J., Majid A., Ukropcová B., De Courten B. (2019). The Potential of Carnosine in Brain-Related Disorders: A Comprehensive Review of Current Evidence. Nutrients.

[97] Sasahara I., Fujimura N., Nozawa Y., Furuhata Y., Sato H. (2015). The effect of histidine on mental fatigue and cognitive performance in subjects with high fatigue and sleep disruption scores. Physiol. Behav..

[98] Van Ruitenbeek P., Sambeth A., Vermeeren A., Young S., Riedel W. (2009). Effects of L-histidine depletion and L-tyrosine/L-phenylalanine depletion on sensory and motor processes in healthy volunteers. Br. J. Pharmacol..

[99] Yoshikawa T., Nakamura T., Shibakusa T., Sugita M., Naganuma F., Iida T., Miura Y., Mohsen A., Harada R., Yanai K. (2014). Insufficient Intake of L-Histidine Reduces Brain Histamine and Causes Anxiety-Like Behaviors in Male Mice. J. Nutr..

[100] Ogawa S., Koga N., Hattori K., Matsuo J., Ota M., Hori H., Sasayama D., Teraishi T., Ishida I., Yoshida F. (2018). Plasma amino acid profile in major depressive disorder: Analyses in two independent case-control sample sets. J. Psychiatr. Res..

[101] Hawkins R.A., O’Kane R.L., Simpson I.A., Viña J.R. (2006). Structure of the Blood–Brain Barrier and Its Role in the Transport of Amino Acids. J. Nutr..

[102] Yamakami J., Sakurai E., Sakurada T., Maeda K., Hikichi N. (1998). Stereoselective blood-brain barrier transport of histidine in rats. Brain Res..

[103] Forrest M., Hill M., Kavanagh D.H., Tansey K., Waite A.J., Blake D.J. (2017). The Psychiatric Risk Gene Transcription Factor 4 (TCF4) Regulates Neurodevelopmental Pathways Associated With Schizophrenia, Autism, and Intellectual Disability. Schizophr. Bull..

[104] He Y., Yu Z., Giegling I., Xie L., Hartmann A.M., Prehn C., Adamski J., Kahn R., Li Y., Illig T. (2012). Schizophrenia shows a unique metabolomics signature in plasma. Transl. Psychiatry.

[105] Mossakowska-Wójcik J., Orzechowska A., Talarowska M., Szemraj J., Gałecki P. (2018). The importance of TCF4 gene in the etiology of recurrent depressive disorders. Prog. Neuro-Psychopharmacol. Biol. Psychiatry.

[106] Webhofer C., Gormanns P., Tolstikov V., Zieglgänsberger W., Sillaber I., Holsboer F., Turck C.W. (2011). Metabolite profiling of antidepressant drug action reveals novel drug targets beyond monoamine elevation. Transl. Psychiatry.

[107] Thakkar M.M. (2011). Histamine in the regulation of wakefulness. Sleep Med. Rev..

[108] Koval’Zon V.M. (2013). The role of histaminergic system of the brain in the regulation of sleep-wakefulness cycle. Hum. Physiol..

[109] Torrealba F., Riveros M.E., Contreras M., Valdes J.L. (2012). Histamine and motivation. Front. Syst. Neurosci..

[110] Tashiro M., Mochizuki H., Iwabuchi K., Sakurada Y., Itoh M., Watanabe T., Yanai K. (2002). Roles of histamine in regulation of arousal and cognition: Functional neuroimaging of histamine H1 receptors in human brain. Life Sci..

[111] Esbenshade T.A., Browman K.E., Bitner R.S., Strakhova M., Cowart M.D., Brioni J.D. (2008). The histamine H3receptor: An attractive target for the treatment of cognitive disorders. Br. J. Pharmacol..

[112] Wada H., Inagaki N., Yamtodani A., Watanabe T. (1991). Is the histaminergic neuron system a regulatory center for whole-brain activity?. Trends Neurosci..

[113] Ghi P., Ferretti C., Blengio M. (1995). Effects of different types of stress on histamine-H3 receptors in the rat cortex. Brain Res..

[114] Endou M., Yanai K., Sakurai E., Fukudo S., Hongo M., Watanabe T. (2001). Food-deprived activity stress decreased the activity of the histaminergic neuron system in rats. Brain Res..

[115] Borges R. (1994). Histamine H1 receptor activation mediates the preferential release of adrenaline in the rat adrenal gland. Life Sci..

[116] Moret C., Briley M. (2011). The importance of norepinephrine in depression. Neuropsychiatr. Dis. Treat..

[117] Montoya A., Bruins R., Katzman M., Blier P. (2016). The noradrenergic paradox: Implications in the management of depression and anxiety. Neuropsychiatr. Dis. Treat..

[118] Yanai K., Tashiro M. (2007). The physiological and pathophysiological roles of neuronal histamine: An insight from human positron emission tomography studies. Pharmacol. Ther..

[119] Kano M., Fukudo S., Tashiro A., Utsumi A., Tamura D., Itoh M., Iwata R., Tashiro M., Mochizuki H., Funaki Y. (2004). Decreased histamine H1 receptor binding in the brain of depressed patients. Eur. J. Neurosci..

[120] Ito C., Shen H.-W., Toyota H., Kubota Y., Sakurai E., Watanabe T., Sato M. (1999). Effects of the acute and chronic restraint stresses on the central histaminergic neuron system of Fischer rat. Neurosci. Lett..

[121] Passani M.B., Lin J.-S., Hancock A., Crochet S., Blandina P. (2004). The histamine H3 receptor as a novel therapeutic target for cognitive and sleep disorders. Trends Pharmacol. Sci..

[122] Haas H.L., Sergeeva O.A., Selbach O. (2008). Histamine in the Nervous System. Physiol. Rev..

[123] Krishnan V., Nestler E.J. (2008). The molecular neurobiology of depression. Nat. Cell Biol..

[124] Uchida S., Hara K., Kobayashi A., Otsuki K., Yamagata H., Hobara T., Suzuki T., Miyata N., Watanabe Y. (2011). Epigenetic Status of Gdnf in the Ventral Striatum Determines Susceptibility and Adaptation to Daily Stressful Events. Neuron.

[125] Lohoff F.W. (2010). Overview of the Genetics of Major Depressive Disorder. Curr. Psychiatry Rep..

[126] Mayeux R. (2004). Biomarkers: Potential uses and limitations. NeuroRx.

[127] Strangman G., Culver J.P., Thompson J.H., Boas D.A. (2002). A quantitative comparison of simultaneous BOLD fMRI and NIRS recordings during functional brain activation. NeuroImage.

[128] Zama T., Takahashi Y., Shimada S. (2019). Simultaneous EEG-NIRS Measurement of the Inferior Parietal Lobule During a Reaching Task With Delayed Visual Feedback. Front. Hum. Neurosci..

[129] Shin J., Von Lühmann A., Kim D.-W., Mehnert J., Hwang H.-J., Müller K.-R. (2018). Simultaneous acquisition of EEG and NIRS during cognitive tasks for an open access dataset. Sci. Data.

[130] Vasic N., Wolf N.D., Grön G., Sosic-Vasic Z., Connemann B.J., Sambataro F., von Strombeck A., Lang D., Otte S., Dudek M. (2015). Baseline brain perfusion and brain structure in patients with major depression: A multimodal magnetic resonance imaging study. J. Psychiatry Neurosci..

[131] Hardikar S., Albrechtsen R.D., Achaintre D., Lin T., Pauleck S., Playdon M., Holowatyj A.N., Gigic B., Schrotz-King P., Boehm J. (2020). Impact of Pre-Blood Collection Factors on Plasma Metabolomic Profiles. Metabolites.

