# Supplementary material for: Integration of the Cortical Haemodynamic Response Measured by Functional Near-Infrared Spectroscopy and Amino Acid Analysis to Aid in the Diagnosis of Major Depressive Disorder
Source: Diagnostics (Basel). 2021 Oct 25;11(11):1978. doi: 10.3390/diagnostics11111978 (PMC8617819; doi:10.3390/diagnostics11111978)
Supplement: Supplementary file 1 [file diagnostics-11-01978-s001.zip › diagnostics-1405311-supplementary.pdf]

Supplementary Materials

Table S1. Measurements of amino acids in 25 healthy controls and 25 major depressive disorder subjects.

Group	Glycine	Alanine	Serine	Proline	Valine	Leucine	Isoleucine	Ornithine	Methionine	Histidine	Phenylalanine	Arginine	Citrulline	Tyrosine	Aspartic acid	Glutamic acid	Tryptophan
Controls	277.4	503.6	166.2	220.9	304.5	153.5	88.9	85.9	33.3	103.7	91.3	129.4	34.6	83.4	32.2	100.3	62.8
	(11.0)	(21.9)	(6.4)	(9.8)	(14.9)	(7.7)	(5.3)	(3.8)	(1.8)	(2.6)	(3.7)	(6.2)	(2.4)	(4.9)	(1.8)	(4.6)	(2.4)
Depressed	289.9	502.5	165.4	219.8	288.6	143.7	83.2	86.8	31.6	93.3	88.0	128.0	33.0	75.6	33.5	102.5	57.0
	(11.0)	(21.9)	(6.4)	(9.8)	(14.9)	(7.7)	(5.3)	(3.8)	(1.8)	(2.6)	(3.7)	(6.2)	(2.4)	(4.9)	(1.8)	(4.6)	(2.4)
ANCOVA[a]	$F = 0.6$<br>dF = 1/45<br>$p = 0.427$	$F = 0.0$<br>dF = 1/45<br>$p = 0.971$	$F = 0.0$<br>dF = 1/45<br>$p = 0.928$	$F = 0.0$<br>dF = 1/45<br>$p = 0.937$	$F = 0.6$<br>dF = 1/45<br>$p = 0.454$	$F = 0.8$<br>dF = 1/45<br>$p = 0.369$	$F = 0.6$<br>dF = 1/45<br>$p = 0.453$	$F = 0.0$<br>dF = 1/45<br>$p = 0.873$	$F = 0.4$<br>dF = 1/45<br>$p = 0.512$	$F = 8.1$<br>dF = 1/45<br>**$p = 0.007$**	$F = 0.4$<br>dF = 1/45<br>$p = 0.534$	$F = 0.0$<br>dF = 1/45<br>$p = 0.878$	$F = 0.2$<br>dF = 1/45<br>$p = 0.644$	$F = 1.3$<br>dF = 1/45<br>$p = 0.265$	$F = 0.3$<br>dF = 1/45<br>$p = 0.608$	$F = 0.1$<br>dF = 1/45<br>$p = 0.731$	$F = 3.0$<br>dF = 1/45<br>$p = 0.091$

Mean ± SEM are shown and p-values ≤0.05 are in bold.
[a]All results of ANCOVA with age, gender, and ethnicity as covariates.

Table S2. Medication details.

	n	Dose (mg/day)
Antidepressants		
Selective serotonin reuptake inhibitors		
Escitalopram	2	10.0 ± 0
Fluoxetine	1	20.0
Paroxetine	1	50.0
Sertraline	4	87.5 ± 47.9
Serotonin and norepinephrine reuptake inhibitors		
Venlafaxine	2	112.5 ± 53.0
Noradrenergic and specific serotonergic antidepressant		
Mirtazapine	2	15.0 ± 0
Other antidepressants		
Vortioxetine	2	20.0 ± 0
Combination antidepressants		
Agomelatine & buproprion	1	
Fluoxetine & mirtazapine	1	
Venlafaxine & buproprion	1	
Anxiolytics and sedatives		
Alprazolam	1	0.25
Diazepam	1	3.5
Lorazepam	1	1.0
Antipsychotics		
Quetiapine	4	82.8 ± 88.0
Mood stabilisers		
Sodium valproate	2	250.0 ± 70.7

Table S3. Correlations between integral values and clinical characteristics in major depressive disorder.

	Frontal region integral		Temporal region integral	
	Test statistic	*p*-value	Test statistic	*p*-value
**Integral value**				
Spearman's correlation				
Gender	-0.008	0.973	-0.259	0.210
Family psychiatric history	0.072	0.750	0.235	0.259
Past trauma history	-0.136	0.546	-0.100	0.635
Pharmacotherapy	-0.100	0.658	-0.178	0.394
Ethnicity	-0.126	0.577	-0.174	0.405
Handedness	0.050	0.826	-0.051	0.808
Pharmacotherapy	-0.036	0.866	-0.178	0.394
Pearson's correlation				
Age (years)	-0.344	0.117	0.053	0.800
Education (years)	-0.111	0.622	0.148	0.481
VFT task performance	0.059	0.799	-0.191	0.370
HAM-D score	0.369	0.068	0.196	0.348
Age at MDD onset (years)	0.145	0.519	0.073	0.730
Duration of MDD (years)	-0.465	**0.029**	-0.002	0.993
Duration of untreated MDD (months)	0.079	0.728	0.342	0.102
Fluoxetine equivalent dose (mg/day)	-0.103	0.715	-0.038	0.886
Diazepam equivalent dose (mg/day)	-	-	-0.455	0.699
Chlorpromazine equivalent dose (mg/day)	0.403	0.597	0.924	0.076

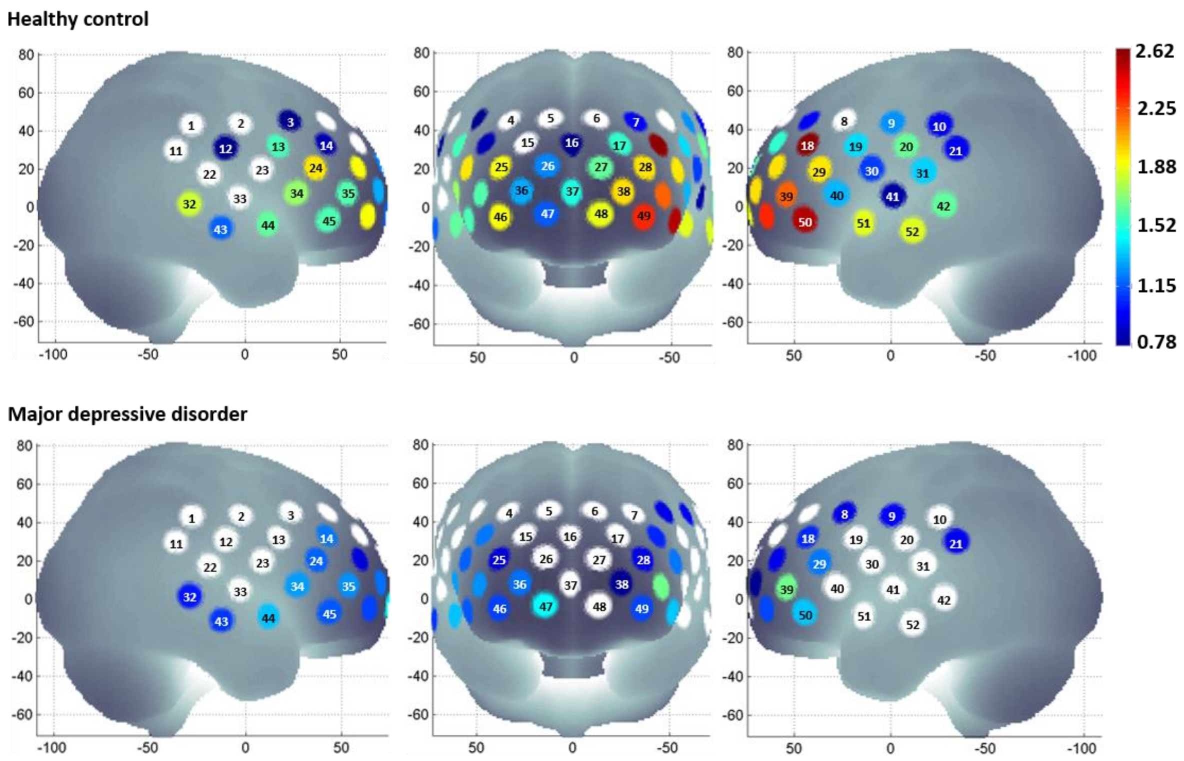

Figure S1. Activation at each channel determined by paired sample t-test comparing the mean oxy-haemoglobin levels during pre-task baseline period and task period. Effect size (Hedge's g) of activation during task period is indicated by the colour gradient. Channels without statistical differences are in white.

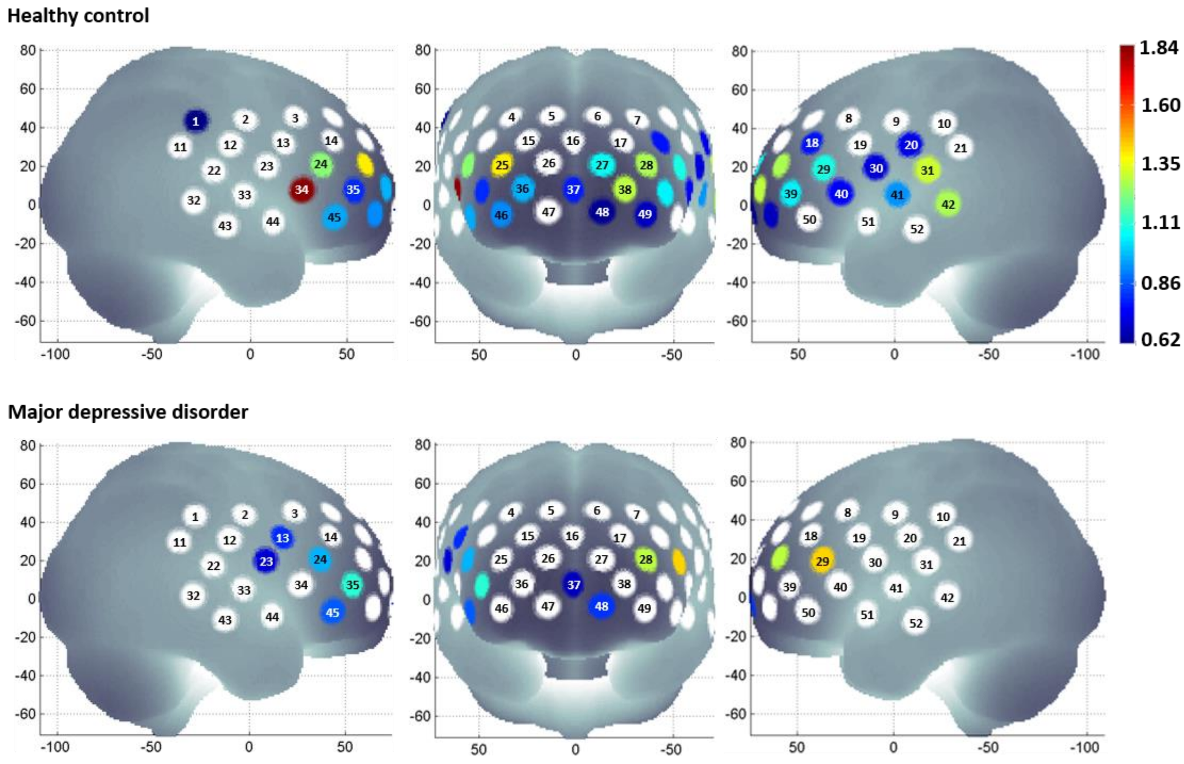

Figure S2. Activation at each channel determined by paired sample t-test comparing the mean deoxy-haemoglobin levels during pre-task baseline period and task period. Effect size (Hedge's g) of activation during task period is indicated by the colour gradient. Channels without statistical differences are in white.

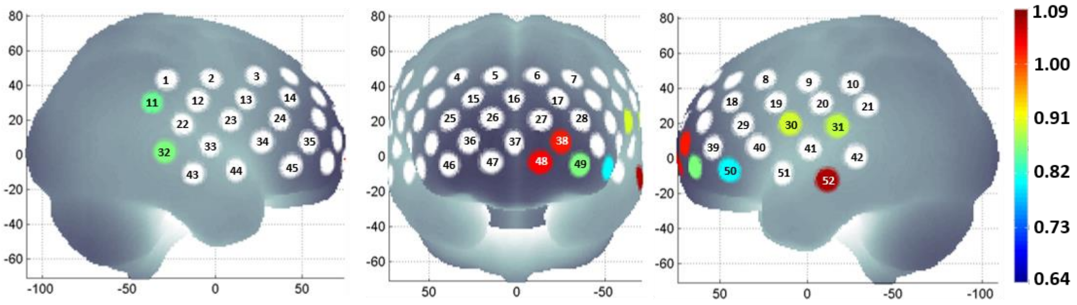

Figure S3. Group differences in mean oxy-haemoglobin levels during task period determined by Student's t-test. Effect size (Hedge's g) of activation during task period is indicated by the colour gradient. Channels with no statistically significant differences are in white.

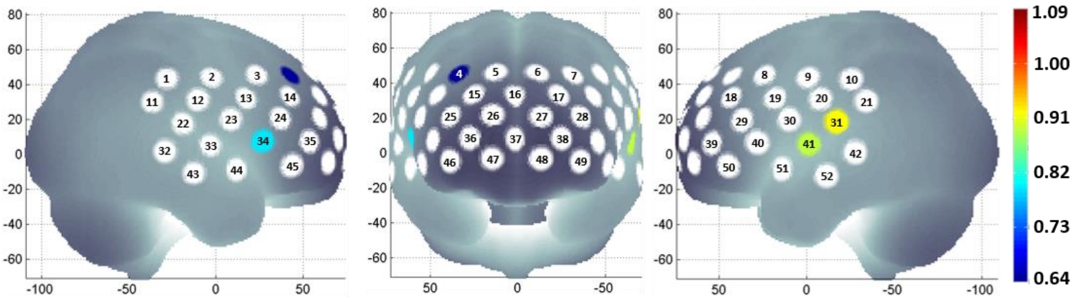

Figure S4. Group differences in mean deoxy-haemoglobin levels during task period determined by Student's t-test. Effect size (Hedge's g) of activation during task period is indicated by the colour gradient. Channels with no statistically significant differences are in white.

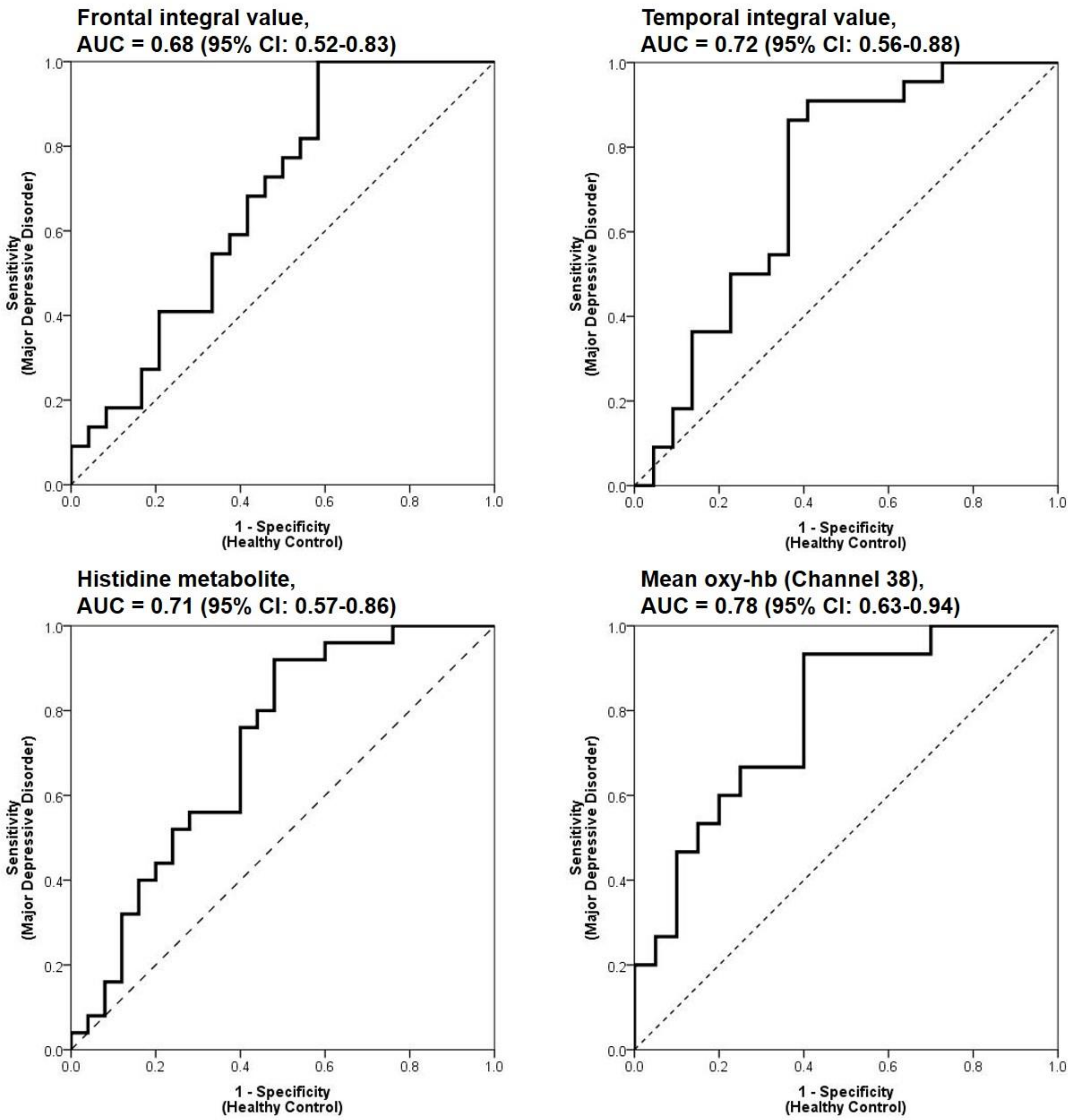

Figure S5. Receiver operating characteristic analysis of single biomarkers between MDD patients and healthy controls.
